# (3*RS*)-*S*-[1-(3-Chloro­phen­yl)-2-oxopyr­rolidin-3-yl]-*N*,*N*′-dimethyl­thio­uronium bromide

**DOI:** 10.1107/S1600536809002153

**Published:** 2009-01-28

**Authors:** Jiří Hanusek, Miloš Sedlák, Pavel Drabina, Aleš Ružička

**Affiliations:** a Institute of Organic Chemistry and Technology, Faculty of Chemical Technology, University of Pardubice, nám. Čs. legií 565, Pardubice 532 10, Czech Republic; b Department of General and Inorganic Chemistry, Faculty of Chemical Technology, University of Pardubice, nám. Čs. legií 565, Pardubice 532 10, Czech Republic

## Abstract

The title mol­ecule, C_13_H_17_ClN_3_OS^+^·Br^−^, consists of benzene and pyrrolidine rings and an S–C(NHCH_3_)_2_ group. The central C—N bond lengths in the S–C(NHCH_3_)_2_ fragment indicate partial double-bond character. Mol­ecules are inter­connected into chains by N—H⋯Br hydrogen bonds and the chains are linked into pairs by weak C—H⋯Br hydrogen bonds.

## Related literature

For the reactivity of the title compound, see: Hanusek *et al.* (2004 ▶); Sedlák *et al.* (2002 ▶, 2003 ▶). For a related structure, see: Hanusek *et al.* (2009 ▶).
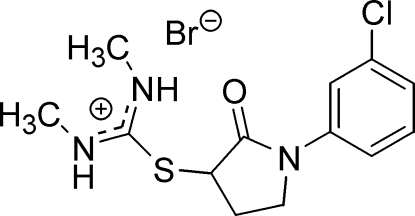


## Experimental

### 

#### Crystal data

C_13_H_17_ClN_3_OS^+^·Br^−^
*M* *_r_* = 378.72
Monoclinic, 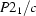

*a* = 14.9409 (9) Å
*b* = 7.7050 (5) Å
*c* = 13.9141 (15) Å
β = 100.758 (7)°
*V* = 1573.6 (2) Å^3^
*Z* = 4
Mo *K*α radiation
μ = 2.91 mm^−1^
*T* = 150 (2) K
0.41 × 0.40 × 0.22 mm

#### Data collection

Bruker–Nonius KappaCCD diffractometer
Absorption correction: gaussian integration (Coppens, 1970 ▶) *T*_min_ = 0.401, *T*_max_ = 0.658
11628 measured reflections
3455 independent reflections
2671 reflections with *I* > 2σ(*I*)
*R*_int_ = 0.060

#### Refinement

*R*[*F* ^2^ > 2σ(*F* ^2^)] = 0.048
*wR*(*F* ^2^) = 0.114
*S* = 1.22
3455 reflections
189 parameters
H atoms treated by a mixture of independent and constrained refinement
Δρ_max_ = 0.74 e Å^−3^
Δρ_min_ = −0.82 e Å^−3^

### 

Data collection: *COLLECT* (Hooft, 1998 ▶) and *DENZO* (Otwinowski and Minor, 1997 ▶); cell refinement: *COLLECT* and *DENZO*; data reduction: *COLLECT* and *DENZO*; program(s) used to solve structure: *SIR92* (Altomare *et al.*, 1994 ▶); program(s) used to refine structure: *SHELXL97* (Sheldrick, 2008 ▶); molecular graphics: *PLATON* (Spek, 2003 ▶); software used to prepare material for publication: *SHELXL97*.

## Supplementary Material

Crystal structure: contains datablocks I, global. DOI: 10.1107/S1600536809002153/fb2123sup1.cif

Structure factors: contains datablocks I. DOI: 10.1107/S1600536809002153/fb2123Isup2.hkl

Additional supplementary materials:  crystallographic information; 3D view; checkCIF report

## Figures and Tables

**Table 1:** Hydrogen-bond geometry (Å, °) *Cg*1 is the centroid of the C5–C10 ring.

*D*—H⋯*A*	*D*—H	H⋯*A*	*D*⋯*A*	*D*—H⋯*A*
N3—H3⋯Br1^i^	0.80 (5)	2.56 (5)	3.314 (4)	159 (5)
N2—H2⋯Br1	0.81 (5)	2.51 (5)	3.303 (4)	170 (5)
C6—H6⋯O1	0.95	2.38	2.899 (5)	114
C8—H8⋯Br1^ii^	0.95	2.87	3.662 (5)	142
C2—H2*A*⋯*Cg*1^iii^	0.99	2.69	3.628 (4)	159

Symmetry codes: (i) 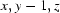; (ii) 
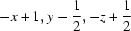
; (iii) 
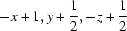
.

**Fig. 1.**
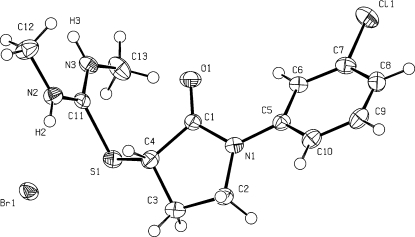
View of the title molecule. The displacement ellipsoids are shown at the 50% probability level. The hydrogens are shown as circles with arbitrary radius.

**Fig. 2.**
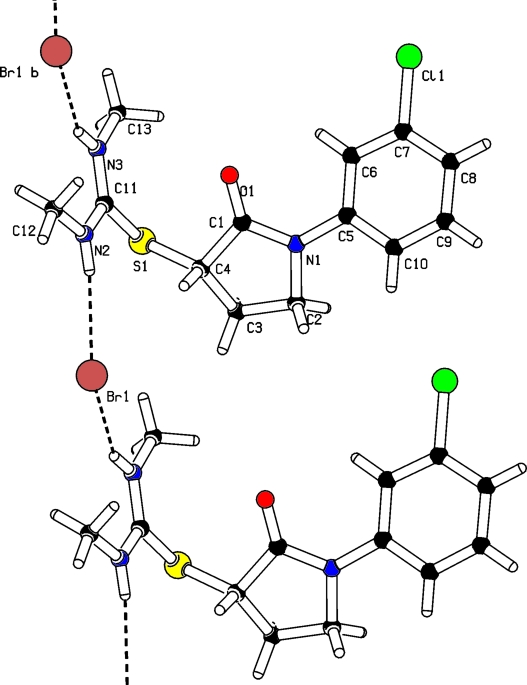
Motif showing the hydrogen bonding in the title structure. Symmetry code for Br1 b: x, 1-y, z.

## supplementary crystallographic information

### Comment

In our previous papers we have discussed the reactivity of the title structure (Sedlák *et al.*, 2002, 2003; Hanusek *et al.*, 2004). In continuation of the above mentioned studies, the related crystal structures of the title compound (Scheme 1, Figs. 1 and 2) as well as of the non-methylated analogue (Hanusek *et al.*, 2009) have been determined and the influence of the N-methyl substituents on the crystal structure has been examined.

The respective important distances for the title compound and its non-methylated analogue are 1.770 (4) and 1.749 (4) Å for S1–C11; (1.307 (5), 1.309 (5) Å) and (1.312 (5), 1.296 (5) Å) for C11–N2 and C11–N3. The respective twist angles about the N1–C5 bonds in the title compound and its non-methylated analogue are 28.8 (2) and 7.8 (1)°.

The interplanar angles between the S—C(NHR)_2_ group and the heterocyclic rings are almost the same in the title compound and its non-methylated analogue (71.3 (1) and 66.7 (1)°). In the S–C(NHCH_3_)_2_ fragment of the title compound, the C-N bond-lengths of N2-C11 and N3-C11 (1.309 (5), 1.307 (5) Å, respectively) indicate a partly double bond character.

All the isothiouronium cations that have been studied in the solid state take part in the hydrogen bonding with different anions. (These anions comprise Cl^-^ as well as complex organic anions.) Also in the title compound and its non-methylated analogue such interactions are present. In the title structure, there is a motif N2–H2···Br1···H1–N1 that links the molecules into the infinite chains parallel to the *b* axis (Fig. 2, Tab. 1). Moreover, the pairs of these chains are interconnected by additional C–H···Br contacts to give columns parallel to the *b* axis. These pairs of the chains are linked by the virtue of two-fold screw axes. There is also a weak C–H···π-electron interaction with π-electrons of the chlorophenyl ring (Tab. 1).

### Experimental

The title compound was synthesized according to Hanusek *et al.* (2004) from saturated acetone solutions of the racemic 3-bromo-1-(3-chlorophenyl)pyrrolidin-2-one and *N*,*N*'-dimethylthiourea. Single crystals (blocks) suitable for analysis were grown directly from the reaction mixture. Their average size was 0.3×0.3×0.2 mm

### Refinement

All the hydrogens were discernible in the difference electron density map, nevertheless they were situated into the idealized positions. Except for for the H(N) that are involved in the hydrogen bonding and therefore their coordinates were refined without constraints or restraints the rest of the hydrogens were refined riding on their parent C: C–H = 0.95, O.98, 0.99, 1.00 Å for the aryl, methyl, methylene and methine hydrogens, respectively. U_iso_(H)=1.2U_eq_(C/N) for the H(N), methylene and methine H atoms, while U_iso_(H)=1.5U_eq_ for methyl hydrogens.

### Crystal data

**Table:**

C_13_H_17_ClN_3_OS^+^·Br^−^	*F*(000) = 768
*M**_r_* = 378.72	*D*_x_ = 1.599 Mg m^−^^3^
Monoclinic, *P*2_1_/*c*	Mo *K*α radiation, λ = 0.71073 Å
Hall symbol: -P 2ybc	Cell parameters from 11663 reflections
*a* = 14.9409 (9) Å	θ = 1–27.5°
*b* = 7.7050 (5) Å	µ = 2.91 mm^−^^1^
*c* = 13.9141 (15) Å	*T* = 150 K
β = 100.758 (7)°	Block, colourless
*V* = 1573.6 (2) Å^3^	0.41 × 0.40 × 0.22 mm
*Z* = 4	

### Data collection

**Table:**

Bruker–Nonius KappaCCD diffractometer	3455 independent reflections
Radiation source: fine-focus sealed tube	2671 reflections with *I* > 2σ(*I*)
graphite	*R*_int_ = 0.060
Detector resolution: 9.091 pixels mm^-1^	θ_max_ = 27.5°, θ_min_ = 2.8°
φ and ω scans	*h* = −19→16
Absorption correction: gaussian integration (Coppens, 1970)	*k* = −8→9
*T*_min_ = 0.401, *T*_max_ = 0.658	*l* = −17→18
11628 measured reflections	

### Refinement

**Table:**

Refinement on *F*^2^	Primary atom site location: structure-invariant direct methods
Least-squares matrix: full	Secondary atom site location: difference Fourier map
*R*[*F*^2^ > 2σ(*F*^2^)] = 0.048	Hydrogen site location: difference Fourier map
*wR*(*F*^2^) = 0.114	H atoms treated by a mixture of independent and constrained refinement
*S* = 1.22	*w* = 1/[σ^2^(*F*_o_^2^) + (0.0325*P*)^2^ + 3.4975*P*] where *P* = (*F*_o_^2^ + 2*F*_c_^2^)/3
3455 reflections	(Δ/σ)_max_ < 0.001
189 parameters	Δρ_max_ = 0.74 e Å^−^^3^
0 restraints	Δρ_min_ = −0.82 e Å^−^^3^
60 constraints	

### Special details

**Table:**

Geometry. All esds (except the esd in the dihedral angle between two l.s. planes) are estimated using the full covariance matrix. The cell esds are taken into account individually in the estimation of esds in distances, angles and torsion angles; correlations between esds in cell parameters are only used when they are defined by crystal symmetry. An approximate (isotropic) treatment of cell esds is used for estimating esds involving l.s. planes.
Refinement. Refinement of F^2^ against ALL reflections. The weighted R-factor wR and goodness of fit S are based on F^2^, conventional R-factors R are based on F, with F set to zero for negative F^2^. The threshold expression of F^2^ > 2sigma(F^2^) is used only for calculating R-factors(gt) etc. and is not relevant to the choice of reflections for refinement. R-factors based on F^2^ are statistically about twice as large as those based on F, and R- factors based on ALL data will be even larger.

### Fractional atomic coordinates and isotropic or equivalent isotropic displacement parameters (Å^2^)

**Table:**

	*x*	*y*	*z*	*U*_iso_*/*U*_eq_	
Br1	0.08338 (3)	0.66896 (5)	0.14195 (3)	0.02613 (13)	
S1	0.16212 (7)	0.32645 (15)	0.36335 (7)	0.0259 (2)	
Cl1	0.62396 (8)	−0.16871 (15)	0.42796 (8)	0.0356 (3)	
N3	0.1148 (2)	0.0146 (5)	0.2861 (3)	0.0225 (7)	
H3	0.095 (3)	−0.053 (7)	0.245 (3)	0.027*	
O1	0.3250 (2)	0.1248 (4)	0.3000 (2)	0.0340 (7)	
C1	0.3345 (3)	0.2769 (5)	0.3210 (3)	0.0219 (8)	
N1	0.4148 (2)	0.3623 (4)	0.3524 (2)	0.0221 (7)	
N2	0.0914 (2)	0.2457 (5)	0.1804 (3)	0.0244 (7)	
H2	0.094 (3)	0.350 (7)	0.178 (4)	0.029*	
C11	0.1189 (2)	0.1804 (5)	0.2677 (3)	0.0207 (8)	
C6	0.5171 (3)	0.1131 (5)	0.3865 (3)	0.0211 (8)	
H6	0.4680	0.0399	0.3948	0.025*	
C7	0.6049 (3)	0.0507 (5)	0.3989 (3)	0.0246 (9)	
C10	0.5754 (3)	0.3920 (6)	0.3476 (3)	0.0261 (9)	
H10	0.5652	0.5102	0.3293	0.031*	
C13	0.1428 (3)	−0.0649 (6)	0.3822 (3)	0.0338 (11)	
H13A	0.2066	−0.0358	0.4080	0.051*	
H13B	0.1362	−0.1912	0.3763	0.051*	
H13C	0.1044	−0.0211	0.4267	0.051*	
C9	0.6624 (3)	0.3230 (6)	0.3608 (3)	0.0278 (9)	
H9	0.7118	0.3945	0.3513	0.033*	
C4	0.2588 (3)	0.4101 (6)	0.3153 (3)	0.0244 (9)	
H4	0.2380	0.4459	0.2456	0.029*	
C2	0.4027 (3)	0.5482 (5)	0.3675 (3)	0.0253 (9)	
H2A	0.4168	0.6166	0.3119	0.030*	
H2B	0.4421	0.5883	0.4286	0.030*	
C5	0.5029 (3)	0.2873 (5)	0.3613 (3)	0.0214 (8)	
C12	0.0567 (3)	0.1404 (6)	0.0946 (3)	0.0341 (11)	
H12A	0.1048	0.0626	0.0814	0.051*	
H12B	0.0372	0.2163	0.0381	0.051*	
H12C	0.0048	0.0716	0.1067	0.051*	
C3	0.3028 (3)	0.5640 (6)	0.3739 (4)	0.0333 (10)	
H3A	0.2770	0.6748	0.3451	0.040*	
H3B	0.2943	0.5575	0.4427	0.040*	
C8	0.6784 (3)	0.1510 (6)	0.3878 (3)	0.0269 (9)	
H8	0.7382	0.1040	0.3983	0.032*	

### Atomic displacement parameters (Å^2^)

**Table:**

	*U*^11^	*U*^22^	*U*^33^	*U*^12^	*U*^13^	*U*^23^
Br1	0.0262 (2)	0.0177 (2)	0.0348 (2)	0.00117 (17)	0.00640 (16)	0.00120 (18)
S1	0.0265 (5)	0.0298 (6)	0.0225 (5)	0.0025 (4)	0.0072 (4)	−0.0046 (4)
Cl1	0.0406 (6)	0.0266 (6)	0.0408 (6)	0.0100 (5)	0.0110 (5)	0.0056 (5)
N3	0.0272 (18)	0.0174 (17)	0.0243 (17)	0.0039 (14)	0.0076 (14)	0.0040 (14)
O1	0.0263 (16)	0.0280 (17)	0.0495 (19)	−0.0037 (12)	0.0118 (14)	−0.0165 (15)
C1	0.020 (2)	0.024 (2)	0.0221 (19)	0.0002 (16)	0.0055 (15)	−0.0043 (17)
N1	0.0251 (17)	0.0195 (18)	0.0217 (16)	−0.0013 (14)	0.0049 (13)	−0.0019 (14)
N2	0.0302 (19)	0.0190 (18)	0.0231 (17)	−0.0002 (15)	0.0030 (14)	0.0019 (16)
C11	0.0189 (18)	0.023 (2)	0.0203 (18)	0.0039 (16)	0.0055 (14)	−0.0034 (17)
C6	0.025 (2)	0.0185 (19)	0.0207 (19)	−0.0008 (15)	0.0072 (15)	−0.0007 (16)
C7	0.032 (2)	0.022 (2)	0.0211 (19)	0.0008 (17)	0.0084 (17)	−0.0003 (17)
C10	0.028 (2)	0.025 (2)	0.025 (2)	−0.0053 (17)	0.0041 (16)	0.0003 (18)
C13	0.040 (3)	0.031 (3)	0.031 (2)	0.013 (2)	0.011 (2)	0.014 (2)
C9	0.027 (2)	0.032 (2)	0.024 (2)	−0.0101 (19)	0.0045 (16)	−0.0005 (19)
C4	0.024 (2)	0.026 (2)	0.023 (2)	−0.0019 (17)	0.0037 (16)	−0.0023 (18)
C2	0.030 (2)	0.018 (2)	0.025 (2)	0.0010 (16)	−0.0010 (17)	0.0004 (17)
C5	0.023 (2)	0.024 (2)	0.0176 (18)	−0.0012 (15)	0.0054 (15)	−0.0028 (16)
C12	0.048 (3)	0.028 (3)	0.023 (2)	−0.003 (2)	−0.0016 (19)	−0.0004 (19)
C3	0.031 (2)	0.027 (2)	0.041 (3)	0.0028 (18)	0.0038 (19)	−0.012 (2)
C8	0.024 (2)	0.038 (3)	0.0196 (19)	0.0029 (18)	0.0055 (15)	−0.0030 (19)

### Geometric parameters (Å, °)

**Table:**

S1—C11	1.770 (4)	C10—C5	1.391 (6)
S1—C4	1.819 (4)	C10—H10	0.9500
Cl1—C7	1.749 (4)	C13—H13A	0.9800
N3—C11	1.307 (5)	C13—H13B	0.9800
N3—C13	1.459 (5)	C13—H13C	0.9800
N3—H3	0.80 (5)	C9—C8	1.385 (6)
O1—C1	1.210 (5)	C9—H9	0.9500
C1—N1	1.367 (5)	C4—C3	1.518 (6)
C1—C4	1.518 (6)	C4—H4	1.0000
N1—C5	1.421 (5)	C2—C3	1.516 (6)
N1—C2	1.464 (5)	C2—H2A	0.9900
N2—C11	1.309 (5)	C2—H2B	0.9900
N2—C12	1.456 (5)	C12—H12A	0.9800
N2—H2	0.81 (5)	C12—H12B	0.9800
C6—C7	1.377 (6)	C12—H12C	0.9800
C6—C5	1.394 (6)	C3—H3A	0.9900
C6—H6	0.9500	C3—H3B	0.9900
C7—C8	1.375 (6)	C8—H8	0.9500
C10—C9	1.385 (6)		
			
C11—S1—C4	98.74 (18)	C10—C9—H9	119.6
C11—N3—C13	125.0 (4)	C8—C9—H9	119.6
C11—N3—H3	122 (4)	C3—C4—C1	104.8 (3)
C13—N3—H3	113 (4)	C3—C4—S1	111.9 (3)
O1—C1—N1	126.8 (4)	C1—C4—S1	112.1 (3)
O1—C1—C4	126.1 (4)	C3—C4—H4	109.3
N1—C1—C4	107.0 (3)	C1—C4—H4	109.3
C1—N1—C5	125.1 (3)	S1—C4—H4	109.3
C1—N1—C2	113.0 (3)	N1—C2—C3	103.7 (3)
C5—N1—C2	121.5 (3)	N1—C2—H2A	111.0
C11—N2—C12	123.3 (4)	C3—C2—H2A	111.0
C11—N2—H2	114 (4)	N1—C2—H2B	111.0
C12—N2—H2	122 (4)	C3—C2—H2B	111.0
N3—C11—N2	122.6 (4)	H2A—C2—H2B	109.0
N3—C11—S1	119.9 (3)	C10—C5—C6	120.4 (4)
N2—C11—S1	117.4 (3)	C10—C5—N1	118.9 (4)
C7—C6—C5	117.7 (4)	C6—C5—N1	120.6 (4)
C7—C6—H6	121.1	N2—C12—H12A	109.5
C5—C6—H6	121.1	N2—C12—H12B	109.5
C8—C7—C6	123.4 (4)	H12A—C12—H12B	109.5
C8—C7—Cl1	118.1 (3)	N2—C12—H12C	109.5
C6—C7—Cl1	118.5 (3)	H12A—C12—H12C	109.5
C9—C10—C5	119.6 (4)	H12B—C12—H12C	109.5
C9—C10—H10	120.2	C2—C3—C4	103.8 (3)
C5—C10—H10	120.2	C2—C3—H3A	111.0
N3—C13—H13A	109.5	C4—C3—H3A	111.0
N3—C13—H13B	109.5	C2—C3—H3B	111.0
H13A—C13—H13B	109.5	C4—C3—H3B	111.0
N3—C13—H13C	109.5	H3A—C3—H3B	109.0
H13A—C13—H13C	109.5	C7—C8—C9	117.9 (4)
H13B—C13—H13C	109.5	C7—C8—H8	121.1
C10—C9—C8	120.9 (4)	C9—C8—H8	121.1

### Hydrogen-bond geometry (Å, °)

**Table:**

*D*—H···*A*	*D*—H	H···*A*	*D*···*A*	*D*—H···*A*
N3—H3···Br1^i^	0.80 (5)	2.56 (5)	3.314 (4)	159 (5)
N2—H2···Br1	0.81 (5)	2.51 (5)	3.303 (4)	170 (5)
C6—H6···O1	0.95	2.38	2.899 (5)	114
C8—H8···Br1^ii^	0.95	2.87	3.662 (5)	142
C2—H2A···Cg1^iii^	0.99	2.69	3.628 (4)	159

Symmetry codes: (i) *x*, *y*−1, *z*; (ii) −*x*+1, *y*−1/2, −*z*+1/2; (iii) −*x*+1, *y*+1/2, −*z*+1/2.
